# Farmacogenética y medicina de laboratorio: del gen individual a los estudios genéticos integrales

**DOI:** 10.1515/almed-2025-0109

**Published:** 2025-09-01

**Authors:** Mercè Brunet Serra, María Isidoro García

**Affiliations:** Sección de Farmacología y Toxicología, Servicio de Bioquímica y Genética Molecular, Centro de Diagnóstico Biomédico, Hospital Clínic Barcelona, Barcelona, España; Instituto de Investigaciones biomédicas August Pi i Sunyer (IDIBAPS), Barcelona, España; Biomedical Research Center in Hepatic and Digestive Diseases (CIBEREHD), Instituto de Salud Carlos III (ISCII), Madrid, España; Departamento de Medicina, Universidad de Barcelona, Barcelona, España; Unidad de Farmacogenómica y Medicina de Precisión, Servicio de Análisis Clínicos y Bioquímica Clínica, Complejo Asistencial Universitario de Salamanca, Salamanca, España; Instituto de Investigación Biomédica de Salamanca IBSA, Salamanca, España; Departamento de Medicina, Universidad de Salamanca, Salamanca, España

## Introducción

En la era de la implementación de la Medicina Personalizada de Precisión, el estudio de los factores genéticos que influyen en la variabilidad de respuesta al tratamiento farmacológico ha demostrado la necesidad de una visión global, que conduce al desarrollo de la farmacogenómica. Este abordaje holístico dirigido a la generación de conocimiento permite elucidar las bases moleculares de la respuesta farmacológica y la búsqueda de nuevas dianas terapéuticas. La farmacogenética, sin embargo, se ha enfocado en el estudio de los biomarcadores de respuesta conocidos y su aplicación en el laboratorio asistencial especializado. En la práctica, ambos términos se emplean en ocasiones indistintamente. En la última década, múltiples grupos de investigación centrados en la identificación y validación de biomarcadores de la respuesta farmacológica han evidenciado la influencia de la genética en la farmacocinética y farmacodinámica de diversos medicamentos [1], [2], [3].

Las sociedades científicas y los consorcios específicos para la implementación clínica en los sistemas nacionales de salud de cada país, han desarrollado documentos con recomendaciones para una prescripción personalizada de fármacos con un grado de evidencia muy elevado de asociación entre farmacogenes y respuesta farmacológica, tanto relativos a toxicidad como eficacia. El Ministerio de Sanidad presentó el Catálogo de Genómica de la *Cartera de Servicios del Sistema Nacional de Salud* (SNS) en junio de 2023, incluyendo un área específica de farmacogenómica [4]. España ha sido el primer país en adoptar una medida para garantizar la equidad en el acceso, aunque el desarrollo de la implementación está siendo desigual en distintas comunidades autónomas [5]. Muchos estudios demuestran el beneficio clínico y económico asociado a las pruebas Farmacogenéticas [6], 7]. Un estudio multicéntrico Europeo [8] demuestra el beneficio clínico de la implementación de las pruebas anticipativas que disminuyen entre un 20–25 % la aparición de las Reacciones Adversas a Medicamentos (RAMs) y optimizan los tratamientos farmacológicos. Sin embargo, las pruebas farmacogenéticas actuales, basadas en el análisis de variantes de genes accionables que nos permiten establecer recomendaciones de prescripción, sólo explican un 20 % de la variabilidad de la respuesta observada en los pacientes. Otros factores como las interacciones fármaco-fármaco, la función hepática y renal, edad, sexo, epigenética, heredabilidad desconocida, y el efecto placebo, entre otros, desempeñan un papel muy relevante en la respuesta individual [9]. Por todo ello, la utilización del Big Data será imprescindible para integrar los datos genéticos, clínicos y ambientales de muchos pacientes, para identificar nuevos biomarcadores farmacogenéticos. La inteligencia artificial permite analizar millones de secuencias genéticas y otras variables asociadas a la respuesta farmacológica. A partir de estos datos, la obtención de algoritmos mediante Machine Learning nos permitirá predecir la respuesta individual a los fármacos y desarrollar sistemas de soporte para su prescripción personalizada.

En esta editorial, evaluamos la situación actual de la farmacogenética en la práctica clínica, sus beneficios y los retos a los que nos enfrentamos en su implementación, desde los aspectos asistenciales a la Salud Digital de Precisión.

## Situación actual

### Enfoque de la farmacogenética

La farmacogenética se puede enfocar atendiendo a distintos criterios, si nos centramos en el espécimen de análisis nos encontramos con estudios en diversos fluidos, (sangre, orina o saliva) o moléculas tisulares como en el contexto oncológico. Si nos centramos en el tipo de variante podemos estar ante una farmacogenética germinal que fundamentalmente indica la respuesta del paciente (por ejemplo según su capacidad de biotransformar o transportar el fármaco) o somática que indica la respuesta del tumor al tratamiento. Según el número de marcadores desde una farmacogenética enfocada en monoterapia (ej. respuesta específica a mavacamten) o en politerapia que sería la farmacogenética en vida real. Si tenemos en cuenta el momento de la determinación, si es previa a la prescripción nos enfrentamos a una farmacogenética anticipativa (genotipos: *DPYD*, *TPMT*) o post prescripción sería una farmacogenética reactiva. También podemos atender a la situación clínica, en el contexto de un trasplante, o la situación evolutiva ante procesos crónicos o agudos, mención especial en este caso a la farmacogenética en emergencia vital: Unidades de Cuidados Intensivos, Unidades de Grandes Quemados o Reanimación, a las que se añade la dificultad del tiempo de respuesta.

### Farmacogenética y proceso analítico

Como en todas las pruebas de laboratorio abordamos el proceso analítico y debemos disponer de un modelo de Gestión de la Calidad preferiblemente centrado en la Norma ISO15189, incluso aunque el laboratorio no esté acreditado. En la Fase Preanalítica atenderemos aspectos como el Consentimiento Informado y el Asesoramiento pre-test, así como la recogida de las variables imprescindibles como son los datos de prescripción, epidemiológicos y clínicos.

En la fase Analítica plantearemos inicialmente que biomarcadores analizaremos en función del enfoque previamente comentado. La cartera del SNS [4] nos puede facilitar la elección, ya que debemos centrarnos en marcadores con alto nivel de evidencia. Respecto a los métodos de análisis, debemos abordar las variables de un solo nucleótido (SNVs) y el número de copias de las variables (CNVs) como en el caso de *CYP2D6* [10]. Podemos optar por estudio simultaneo mediante espectrometría de masas (LC/MS) o secuenciación masiva o secuencial combinando distintas técnicas basadas en la reacción en cadena de la polimerasa con transcriptasa inversa (RT-PCR), *arrays* o *sanger*, sin embargo, es necesario disponer de sistemas de confirmación como la PCR digital.

En la fase postanalítica, debemos prestar atención a la elaboración del informe que debe basarse en recomendaciones de Guías y cumplir todos los requisitos de la Norma 15189, ser lo más completo posible incluyendo si disponemos de información sobre epigenética, fisiopatología del paciente, hábitos, medicación concomitante, interacciones fármaco-fármaco y fármaco-gen. La comunicación de resultados y correspondientes recomendaciones farmacogenéticas debe estar realizado por personal facultativo con experiencia.

### Implementación

Por lo que respecta a la implementación de la farmacogenética, la situación es claramente desigual dependiendo de las comunidades autónomas, sin embargo, la cartera del SNS proporciona un marco que favorece la equidad y permite una homogeneización en la atención. Tenemos algunos modelos de éxito, como el modelo *Five Step Precision Medicine* 5SPM (5SPM) en Castilla y León que analiza en vida real la prescripción completa del paciente y en la actualidad incluye digitalización e implementación de la Inteligencia Artificial (IA); el proyecto Medea en Extremadura para el farmacogenotipado de la población; el proyecto de Salud Mental de la Fundación Xenómica en Galicia o la Iniciativa Farmacogenética de la Comunidad de Madrid. Actualmente, se están implementando modelos en otras comunidades, entre ellas Valencia y Cataluña. Esta última ya dispone del Programa de oncología de precisión en el Sistema Sanitario Público.

## Retos de futuro

La *formación* es un reto fundamental que no se aborda en profundidad en las facultades, y se debe complementar durante la especialización, mediante formación continuada o rotaciones específicas que aseguren la adquisición de las competencias necesarias en los ámbitos analítico, tecnológico, bioinformático, científico o farmacológico.

Nos encontramos ante un proceso complejo en el que intervienen múltiples factores, muchos aún desconocidos. La *investigación* es esencial en la generación del conocimiento sobre las bases moleculares que permitan identificar nuevos marcadores y dianas terapéuticas. El papel de la multiómica resulta fundamental desde la farmacoexposómica orientada al estudio sistemático de los factores exógenos y su asociación con la respuesta farmacológica, la farmacoepigenómica enfocada hacia los factores epigenéticos que influyen en la respuesta a los fármacos, la farmacotranscriptómica revisando los perfiles globales de expresión de RNA relacionados con la respuesta, la farmacoproteómica que analiza los perfiles de expresión proteicos en el contexto del tratamiento farmacológico; o incluso la metabolómica que incluye el análisis global de los metabolitos implicados; en definitiva, la aplicación de la Biología de Sistemas a la investigación traslacional y su trasferencia en la innovación.

La *ordenación asistencial* adquiere gran importancia, especialmente en atención primaria que dispone de una visión completa del abordaje terapéutico y que permite un enfoque de farmacogenética en vida real. En atención hospitalaria, debemos tener en cuenta el contexto vital para analizar con rapidez marcadores precisos, con nivel de evidencia adecuado y, si es posible, con carácter anticipativo. Además, la elevada especialización hospitalaria no debe perder de vista la visión global del paciente lo que subraya la importancia del equipo multidisciplinar.

Uno de los retos más importantes es la *financiación*, sin dotación económica nada de esto es posible. Los recursos humanos resultan determinantes, se precisan profesionales altamente cualificados. Respecto a las infraestructuras de alto rendimiento se están desarrollando estudios de secuenciación de nueva generación (NGS), inicialmente secuenciación completa del exoma (WES) y actualmente secuenciación completa del genoma (WGS). Sin embargo, la complejidad técnica, el desconocimiento del impacto de las nuevas variantes, la escasa estandarización y validación así como la dificultad en el proceso de acreditación suponen importantes retos para el laboratorio.

Los profesionales especializados debemos dar respuesta a las necesidades actuales para aplicar eficientemente la farmacogenética. La trasformación de la medicina tradicional en Medicina Personalizada de Precisión (MPP) está condicionada por las “ómicas” por lo que el *laboratorio de precisión* adquiere un papel fundamental en el manejo de infraestructuras complejas. La NGS nos enfrenta al *Big Data* que requiere la aplicación de la IA así como grandes instalaciones de almacenamiento. Además, el informe farmacogenético debe elaborarse por expertos teniendo en cuenta la información analítica y aspectos concomitantes que le imprimen un carácter dinámico que puede precisar reevaluaciones posteriores y modificación en las recomendaciones. El reto de la revolución del laboratorio requiere un abordaje multidisciplinar en el que el profesional de laboratorio adquiere un papel consultor preeminente, motor de la transición de las especialidades de precisión.

Esta gran revolución de la farmacogenética en la MPP no se entendería sin los grandes avances en *salud digital de precisión* además de los mencionados *Big Data* e IA, destacamos, el control de los flujos, la interoperabilidad, la integración con el Sistema Informático de Laboratorio (SIL), el software de interpretación o, considerando el carácter multifactorial de la respuesta farmacológica, la integración de información procedente de la telemedicina y de dispositivos portátiles de monitorización o *wearables*.

Finalmente nos encontramos retos éticos y legales. La tecnología avanza más deprisa que el marco legislativo si bien el abordaje de las agencias reguladoras a través de las fichas técnicas [11] nos facilita la aplicación de la farmacogenética. Por último, destacar el reto social al afrontar un cambio cultural y especialmente el compromiso personal, que como profesionales nos debemos preguntar si estamos dispuestos a asumir.

## Conclusiones

La Medicina de Laboratorio evoluciona con la MPP. La farmacogenética aporta nuevas oportunidades para incrementar la seguridad y eficacia de los medicamentos, con un claro beneficio clínico y económico para el SNS. Se requiere una estandarización de los procesos analíticos y de los informes farmacogenéticos. Considerando la complejidad y todos los aspectos con influencia en la variabilidad interindividual de la respuesta farmacológica, es imprescindible una capacitación altamente especializada de nuestros facultativos y una evolución de nuestro laboratorio hacia un laboratorio de precisión.
